# Clinical validation of software for a versatile variability analyzer: Assessment of autonomic function

**DOI:** 10.4103/0971-6203.35721

**Published:** 2007

**Authors:** T. S. Ananthakrishnan, G. D. Jindal, Vineet Sinha, Rajesh K. Jain, S. K. Kataria, Alaka K. Deshpande

**Affiliations:** Electronics Division, Bhabha Atomic Research Centre, Mumbai, India; * Raja Ramanna Fellow, IIT, Mumbai, India; ** Department of Medicine, JJ Hospital, Mumbai, India

**Keywords:** Blood pressure variability, medical analyzer, physiological variability, heart rate variability

## Abstract

Study of physiological variability is an upcoming area of research having manifold clinical applications. Considerable work has been done on heart rate variability and blood pressure variability during the past four decades. Electronics division, Bhabha Atomic Research Centre, has developed an instrument called medical analyzer, which can be used to study several variabilities simultaneously. This instrument has been used to collect data from control subjects and patients with established diagnosis. The data has been analyzed with the help of a software package developed for this purpose and has been found to be consistent with expected manifestations of the disease on the autonomic nervous system. The description of the software package and results of the study are briefly described in this paper.

Nerves, which predominantly participate in the regulation of bodily functions, are covered by the autonomic nervous system. These nerves generally function without consciousness or volitions. These are further divided into two categories - namely, sympathetic nerves and parasympathetic nerves. Both these classes have complementary effect on the body function, such as exciting the heart, constricting blood vessels, decreasing gastrointestinal motility and constricting sphincters, etc., by sympathetic nerves and opposite responses by the parasympathetic nerves.

Due to ease of recording of the activities of the heart, majority of the studies on autonomic functions are focused on the cardiovascular system.

Sayers,[1] Akselrod *et al.*[2] and others have analyzed the frequency contents of these heart rate fluctuations by power spectrum analysis as a pioneering work into the investigations of heart rate variability (HRV). They have shown that in the power spectrum of heart rate fluctuation, there are three dominant peaks typically centered around 0.04, 0.12 and 0.4 Hz respectively as shown in Figure 1a. These peaks are labeled as low-frequency (LF) peak, mid-frequency (MF) peak and high-frequency (HF) peak accordingly. The high-frequency peak centered on 0.4 Hz represents well-known fluctuations in the heart rate associated with the respiratory cycle. The origin of low-frequency peak and mid-frequency peak was suggested to be related to cyclic fluctuations in peripheral vasomotor tone associated with thermoregulation and response of the baro-recepter reflex respectively.[3] The power spectral density after giving parasympathetic blocking agent and total autonomic blocking agent in the same subject is shown in Figure 1b. It can be seen that after administration of glycol-pyrrolate, the amplitude of LF peak decreases and that of MF peak and HF peak reduces nearly to zero. The complete autonomic blockade abolishes all the three peaks as shown by the dotted line in the figure.

**Figure 1:**
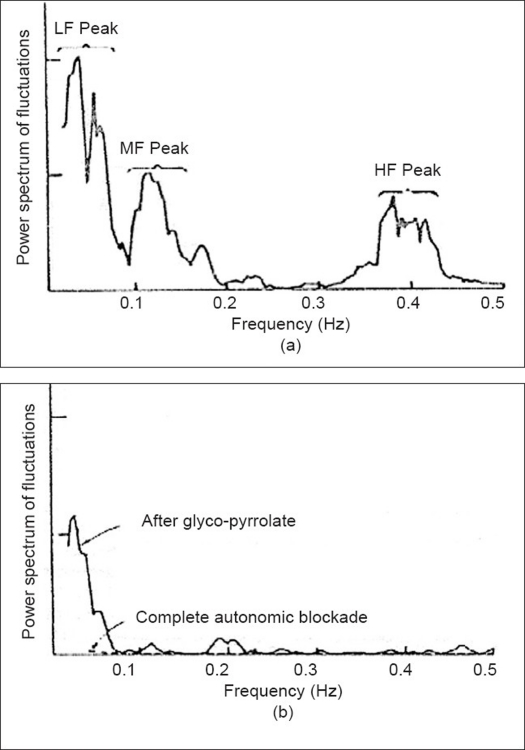
(a) Power spectral density of variations in the heart rate in an adult conscious dog. (b) The power spectral density after giving parasympathetic blocking agent and total autonomic blocking agent in the same subject (courtesy, Akselrod *et al*.)

During the study of effect of a pharmacological agent on microcirculation in normal subjects, Jindal *et al.*[4] recorded variations in the peripheral blood flow in the lying-down subjects before and after the administration of a vasodilator. It was surprising to observe that 80-90% variation in peripheral blood flow took place even before the administration of the drug. To identify the cause of these variations, they recorded similar data in 27 healthy volunteers and 15 patients with hypertension/ diabetes. Figures 2a and 2b show blood flow data recorded from a control subject and a patient with systemic hypertension. The peripheral blood flow exhibits marked variations from beat to beat in the normal subject in contrast to that observed in the patient.

**Figure 2:**
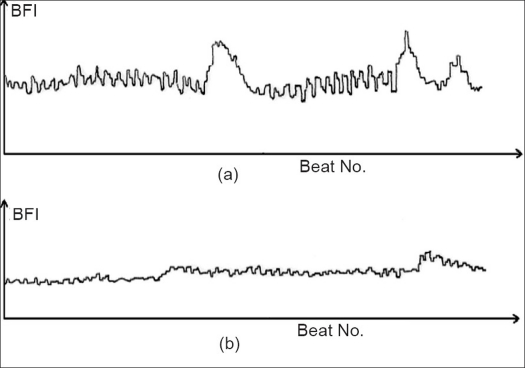
Blood flow index per cardiac ejection plotted against the heartbeat number recorded by impedance plethysmograph in (a) a normal subject and (b) a hypertensive patient in resting condition

The authors have developed a versatile system called the medical analyzer for recording variability patterns in a number of physiological parameters such as heart rate (HR), stroke volume (SV), cardiac output (CO) and peripheral blood flow (PFB) using the principle of impedance plethysmography (IPG). The application software and its clinical validation are briefly described in this paper.

## Materials and Methods

The medical analyzer developed in Electronics Division, BARC, is based on the principle of IPG. In this system, the electrical impedance (Z) of the thoracic region or wrist is measured by the constant-current method. Blood volume changes in the body segment cause minor changes in the electrical impedance, which are used to estimate the blood flow in the respective segment.[5]

**Figure 3:**
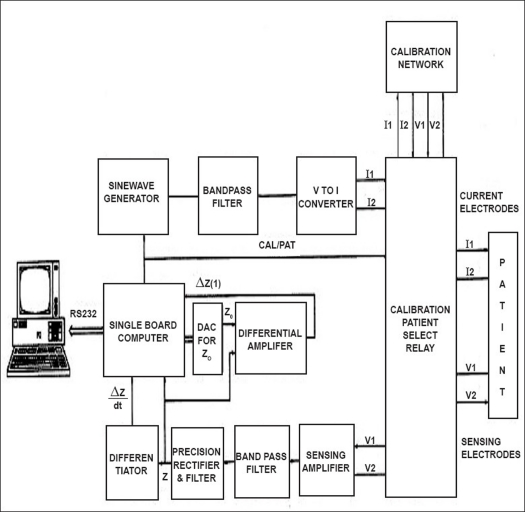
Block diagram of medical analyzer system developed at BARC

Figure 3 shows basic block diagram of the medical analyzer system developed at Electronics Division, Bhabha Atomic Research Centre. It is comprised of an ‘Erasable programmable read-only memory (EPROM)’-driven sine wave generator producing sine waves at 50 KHz frequency. The control input for the sine wave generator for different modes comes from the single board computer (SBC) as shown in the figure. The sine wave generator is followed by a band pass filter (Q > 10) for deglitching the sine wave output. The output of the band pass filter is fed to a voltage-to-current converter, which allows passage of sine wave current of constant/modulated amplitude through the patient calibration network. The signal developed along the current path is sensed with the help of electrodes V1 and V2 and is amplified using a differential amplifier. The amplified signal is rectified and low-pass filtered to obtain the voltage signal Z, which is directly proportional to the electrical impedance of the body segment between sensing electrodes. Z is differentiated and amplified for obtaining rate of change of impedance (dZ/dt), which is connected to the SBC for digitization and further processing. The Z signal is also fed to another differential amplifier for obtaining change in impedance as a function of time [ΔZ(t)]. The non-inverting input of this differential amplifier is obtained from a 12-bit digital-to-analog converter (DAC), which outputs the initial value of impedance (Zo). The DAC derives its input from output ports of the single-board computer so that minor adjustments in the value of Zo can be made through PC. The signals Z and δZ(t) are also connected to remaining analog-to-digital converter (ADC) inputs of the SBC. The SBC is connected to a personal computer through a serial link (RS 232 C) as shown in the figure. Thus the PC performs the operation of the instrument, as well as the data acquisition (patent, 5).

**Figure 4:**
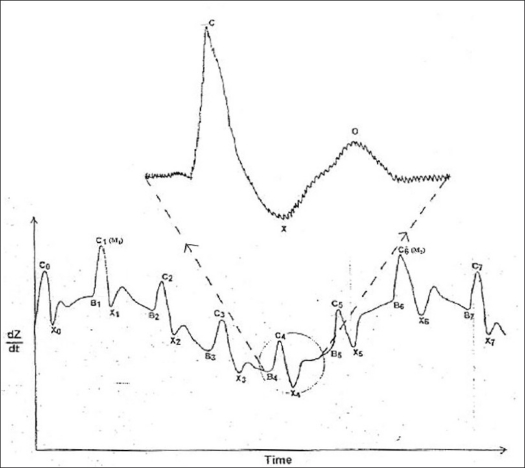
Normalized thoracic impedance plethysmogram recorded from a subject

Figure 4 shows typical thoracic impedance plethysmogram as a function of time recorded from a subject. It shows positive peaks occurring rhythmically with an approximate period of 0.8 s, which are marked as C_o_, C_1_, C_2_………C_7_ in the figure. These peaks are caused by rhythmic ventricular contractions and represent blood flow out of the heart. One of the peaks, namely, C_4_, is zoomed and displayed in the same figure. The height BC normalized with respect to basal impedance gives the proportional value of stroke volume. Wandering of the base line is also observed in the waveform shown in the figure, which is caused by the respiration of the subject and can be used to derive respiratory parameters if required.

Since BCX (portion shown in Figure 4) complex is consequent to the ventricular contraction, consecutive BCX complexes can be used to determine the instantaneous heart rate in a manner similar to that in electrocardiogram. Therefore, after the data acquisition, C points are detected automatically by searching maxima in the neighborhood of 60 to 120 samples and marked as C_o_, C_1_, C_2_……….C_n_ in the entire data. Manual correction, if required, can be made through the application program in order to account for motion artifacts, etc., by scanning the data manually. Multiplication of SV by HR gives the proportional value of CO.[6] Thus single data acquisition from thoracic region can be used to derive variabilities in HR, SV and CO. Similar measurements in peripheral impedance plethysmogram give the proportional value of blood flow index as a function of time.[7] Frequency domain representation of these values then yields the periodicities in these variables. Derivations of these variables are depicted in the following equations.

According to Kubicek's formula, SV is given as:

$${\text{SV=ρL}}^{2}{\text{ T}}_{\text{LVET}}\;{\left(\text{dZ/dt}\right)}_{\text{m}}/{\text{Z}}_{0}^{2}\;\mathrm{(1)}$$

where ρ is the resistivity of blood in Ω-cm, L is the length in cm between sensing electrodes, T_LVET_ is the left ventricular rejection time in seconds and (dZ/dt)_m_ is the maximum height of IPG waveform in ohm/s, as depicted in Figure 4, and Z_o_ is the basal impedance value in ohms.

For the purpose of variability study, Equation 1 can be simplified by taking ρ = 135 Ω-cm, L/Z_o_ = 1.0 and T_LVET_ =0.3 s as:

$$\text{SV=40}{\text{.5}}^{*}{\text{ (dZ/dt)}}_{\text{m}}\;\mathrm{(2)}$$

Equation 2 can be used to determine beat-to-beat SV (SV_i_) at time instant t_i_ for the study of variability.

For obtaining beat-to-beat CO_i_ in liters/min, SV_i_ can be multiplied by heart rate as follows:

$${\text{CO}}_{\text{i}}={\text{SV}}_{\text{i}}*{\text{ RR}}_{\text{i}}/16.67\;\mathrm{(3)}$$

After obtaining instantaneous values of HR, SV, CO and PBF, the data is interpolated using linear techniques to obtain the values of these parameters at equal intervals of time. The fast Fourier transform (FFT) of the interpolated data gives the power spectral density (PSD) of the variability of physiological parameter. Thus it is possible to depict various rhythms responsible for fluctuation in the values of a parameter.

### Application software

The application software developed for the study of variability has been focused on the data acquisition from the patient and detection of different variability rhythms in various physiological parameters. For the short-term studies (typically 5 min) planned in this system, the number of measurable parameters for a single subject is too large. For instance one can study HR variability, SV variability, CO variability and PBF variability (right/ left) in a subject. Each of this variability is characterized by base line, total power and up to six rhythms in different frequency bands. Each of these rhythms is further characterized by center frequency, left cutoff, right cutoff, maximum amplitude and area under the peak. Thus the total number of measurable parameters turns out to be 160 per subject. Analysis of this data in large number of subjects necessitates employment of database utility.

**Figure 5:**
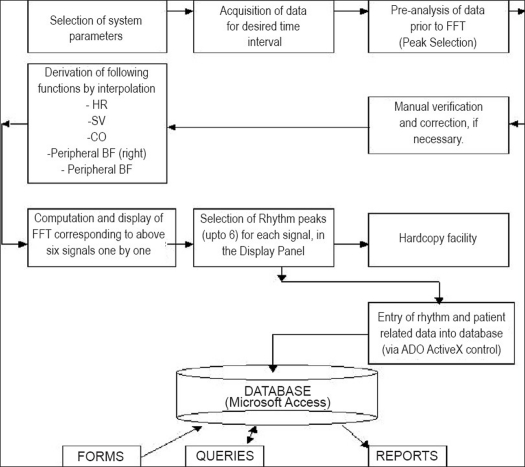
System operation scheme

Figure 5 shows an overview of the application software with the help of operation sequence schematic. With the system ‘ON,’ parameter and lead are first selected for acquiring the data for desired interval of time. At the end of data acquisition, the data is saved and then we enter into the pre-analysis phase, i.e., selection of important points in the data, also known as ‘peak selection.’ In this phase, the first relevant ‘C’ pt. is marked manually and all subsequent similar points are detected automatically in time, as well as in amplitude domain. These points are manually verified. In case of error detection of one or few points, facility for manual correction is provided. A composite display of all the selected points is also available on the screen to quickly point out any anomaly. This data is then used to derive the heart rate, stroke volume and cardiac output signals as function of time with suitable interpolation techniques in case of chest lead and peripheral blood flow (right/ left) in case of limb lead. This input data is then used to obtain power spectral density by using FFT and displayed one by one. The rhythms of variability in the power spectral density are then manually selected up to a maximum of six in the frequency range of 0.01-0.50 Hz. The processed power spectral density, along with patient-related information, is either printed or entered into the database via ActiveX Data Objects (ADO) control. Database generates reports depending upon the queries fired.

### System function

**Figure 6:**
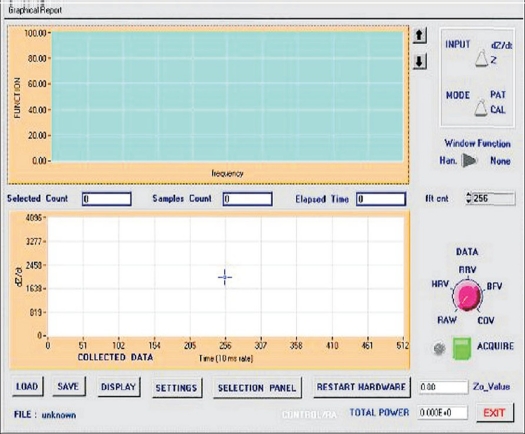
Main panel

On startup, a user-friendly virtual instrumentation control panel appears on the monitor. This is the main panel and is given in Figure 6. The graph on the upper side is for display of power spectral density, and input data to FFT is displayed in the lower graph. The data to be displayed on the upper and lower graph is selectable by a band switch named ‘data’ with selections corresponding to RAW, HRV, blood flow variability (BFV) and CO variability. Data acquisition is also done from the same panel. To start with, ‘mode’ is selected as CAL and input as Z or dZ/dt from the box at the upper right corner and ‘Acquire’ button is clicked. A straight line or a square wave is observed, depending upon input selection. After observing satisfactory calibration signal, the system is switched into patient mode. To enter personal details of the patient, one has to go to another panel - ‘SETTINGS.’

### Data acquisition

The data acquisition is started by clicking on the ‘Acquire’ push button in the main panel. An LED lamp indication shows that the data acquisition is ‘ON.’ A timer control set for 10-ms intervals will trigger a handler every 10 ms, which will collect data from the medical analyzer hardware and display the data on screen after saving the data in buffer. The data acquisition is stopped either on the completion of set time or on pressing the same button that was used to start the acquisition.

A command button ‘SAVE’ has to be pressed to save the data. If the user of the system forgets to save the data and closes the system, a confirmation popup window will remind the user to save the data. The data is saved with a header followed by the data itself. The file name is being generated by proper concatenation of group name, name of the patient and the lead from where acquisition has been done. Also, using the ‘LOAD’ button, it is possible to load a saved file for display/analysis.

### Data processing

**Figure 7:**
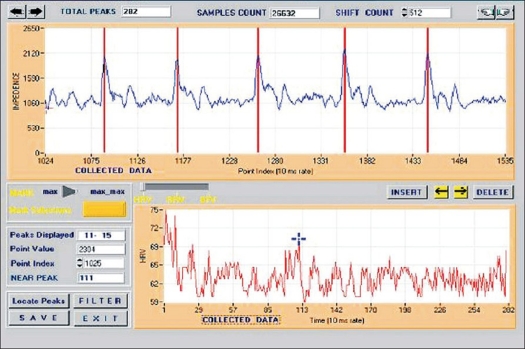
Data selection panel

The data consists of several peaks of different heights (like C_i_ in Figure 4) occurring rhythmically. Also, it is necessary to identify the correct peaks. Figure 7 shows the picture of the selection panel. Generally, the peaks are well defined except when some artifacts occur. Once the peaks (maxima) are displayed on the graph (upper graph), one can start peak selection. It is possible that some initial portion of the data may have to be skipped. That is, a provision is made to locate peaks from a user-selectable starting point in the data. (Acquired data is referred to as raw data). Subsequent peaks are located automatically by the application with programmable search criteria. Composite data of selected peak points appear in the tabular box provided at the right bottom corner in the same panel in time domain. Any anomaly can be easily visualized on this graph and can be subjected to manual correction with navigating buttons provided on the screen.

The selected points are saved in the same file, and then the data is subjected to interpolation using Spline algorithm to make the same periodic. The interpolated data is subjected to Fourier Transform using FFT algorithm.

**Figure 8:**
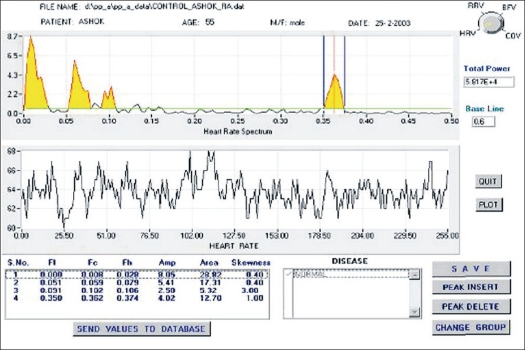
Display panel showing heart rate variability in a control subject

Clicking the display button in the main panel gives ‘display panel,’ as shown in Figure 8. Here the selected data is displayed (in the lower graph), along with FFT (upper graph). This panel is used for feature extraction for each variability and generation of hard copy. The data that can be selected for display are HRV, SV variability/ BFV and CO variability. For each of the variabilities displayed, it is required to identify peaks in different frequency ranges in the FFT display. For each peak identified, attributes like peak amplitude, center frequency, left frequency cutoff, right frequency cutoff, area under the peak and skewness of peak are to be measured. For this purpose, cursors have been provided. Cursors are placed suitably on left cutoff, right cutoff and center of the peak. Subsequently, on pressing the insert button, the features of the selected peak are inserted into a list below the FFT graph. Up to six peaks can be taken into account for a given parameter. These values can be directly sent to ‘Microsoft Access’ database, which is used for generation of various queries and reports. After completing the extraction of features from the FFTs, by clicking on the button labeled ‘plot,’ one can get a screen printout.

## Results and Discussion

**Figure 9:**
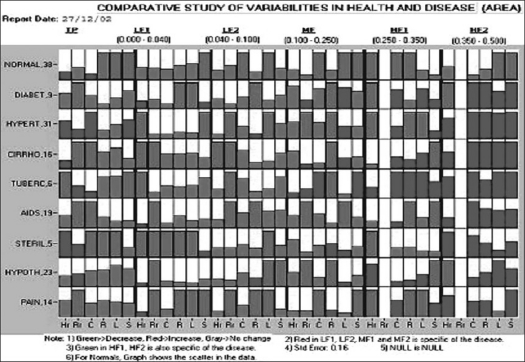
Graphical representation of selective behavior of physiological variabilities in different diseases

Using this system, data has been acquired from 38 normal subjects, 9 patients with diabetes, 31 patients with hyperthyroidism, 16 patients with cirrhosis of liver, 6 patients with pulmonary tuberculosis, 19 patients having human immunodeficiency virus positive, 5 patients with sterility, 23 patients with hypothyroidism, 14 patients with joint pains and 50 patients with mixed disorders. The diagnosis in these patients was established in the respective departments of JJ hospital. It is observed that total power, low-frequency variations and medium-frequency variations are decreased, and high-frequency variations are increased in general in all the diseases. However, selected parameters in selected frequency range show anomalous behavior to specify the disease as shown in Figure 9. Red areas in TP, LF1, LF2 and MF and green areas in HF1 and HF2 are therefore specific of the disease. The Table 1 shows the relation between diseases and variabilities as obtained by the authors.

**Table 1:** Relation between diseases and variabilities

*Disease*	*Increase in total power*	*Increase in LF (Area)*	*Increase in MF (Area)*	*Decrease in HF*
Diabetes	-	HRV, SSV	RRV, PBFVL	PBFVR
Hyperthyroid	HRV, RRV, COV, SSV	PBFVR	PBFVL	-
Cirrhosis	HRV	-	RRV	HRV
Tuberculosis	RRV, COV	-	HRV, RRV, COV, SSV	-
Acquired immunodeficiency syndrome	-	HRV	-	HRV, PBFVL
Sterility	HRV, RRV	SSV	PBFVL, PBFVR	
Hypothyroid	RRV	HRV, RRV	PBFVL	-
Pain	-	HRV	PBFVR	PBFVL

**Figure 10:**
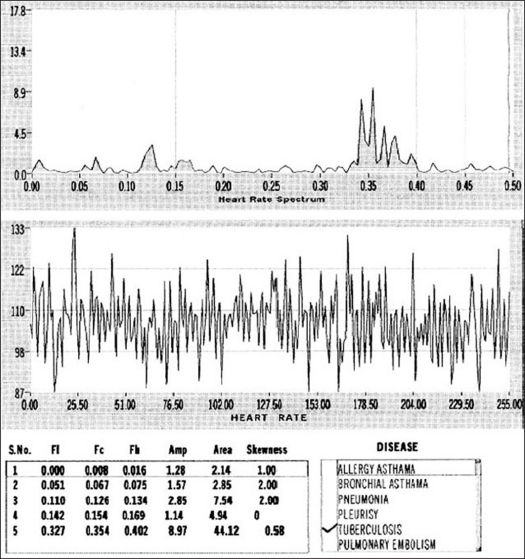
Typical heart rate fluctuations in time (lower) and frequency domain (upper) in a patient suffering from pulmonary tuberculosis

Figure 10 illustrates HRV in a patient with pulmonary tuberculosis. As can be seen from this figure, LF and MF peaks are suppressed and HF peak is enhanced, making this pattern different from that in normal subjects [Figure 8]. Other diseases also have given specific variability pattern and show potential of using physiological variability for disease characterization. Though HRV pattern recorded by our system and that obtained from standard package on the same individual subject are not available, gross comparison of these patterns shows no significant differences.[8,9] The results of this study show the usefulness of this technique in disease characterization and therefore validate the software package developed by us.

To date some attempts have been made to record variability patterns in heart rate and blood pressure, which have been correlated to the autonomic function of the subjects. The potential of HRV has been shown to be so great that now it is a common feature in all the monitoring instruments and is used to see prognosis in patients after the infarction. It is for the first time an instrument and software package has been developed which not only gives heart rate variability but also variability in SV, CO and PBF from the same physiological signal, thereby extending to other physiological parameters. A detailed study on large number of patients with variety of diseases is in progress, which may validate this technique as a potential diagnostic tool.
